# Recognizing Intimate Partner Violence in Primary Care: Western Cape, South Africa

**DOI:** 10.1371/journal.pone.0029540

**Published:** 2012-01-05

**Authors:** Kate Joyner, Robert Mash

**Affiliations:** 1 Division of Nursing, Faculty of Health Sciences, Stellenbosch University, Tygerberg, South Africa; 2 Division of Family Medicine and Primary Care, Faculty of Health Sciences, Stellenbosch University, Tygerberg, South Africa; The University of Queensland, Australia

## Abstract

**Introduction:**

Interpersonal violence in South Africa is the second highest contributor to the burden of disease after HIV/AIDS and 62% is estimated to be from intimate partner violence (IPV). This study aimed to evaluate how women experiencing IPV present in primary care, how often IPV is recognized by health care practitioners and what other diagnoses are made.

**Methods:**

At two urban and three rural community health centres, health practitioners were trained to screen all women for IPV over a period of up to 8 weeks. Medical records of 114 thus identified women were then examined and their reasons for encounter (RFE) and diagnoses over the previous 2-years were coded using the International Classification of Primary Care. Three focus group interviews were held with the practitioners and interviews with the facility managers to explore their experience of screening.

**Results:**

IPV was previously recognized in 11 women (9.6%). Women presented with a variety of RFE that should raise the index of suspicion for IPV– headache, request for psychiatric medication, sleep disturbance, tiredness, assault, feeling anxious and depressed. Depression was the commonest diagnosis. Interviews identified key issues that prevented health practitioners from screening.

**Conclusion:**

This study demonstrated that recognition of women with IPV is very low in South African primary care and adds useful new information on how women present to ambulatory health services. These findings offer key cues that can be used to improve selective case finding for IPV in resource-poor settings. Universal screening was not supported by this study.

## Introduction

In South Africa interpersonal violence is the second highest contributor to the burden of disease, after HIV/AIDS [1]. Interpersonal violence, therefore, contributes more to the burden of disease than common diseases such as tuberculosis, pneumonia, gastroenteritis, hypertension or depression. Intimate partner violence (IPV) accounts for 62.4% of the total interpersonal violence burden in females. More women are killed in South Africa by their current or ex-intimate male partner than in any other country with a rate of 8.8 per 100000 women [2].

The Domestic Violence Act of 1998 defined IPV as “actual or threatened physical, emotional, verbal, sexual and/or financial abuse” [3]. The intent, and sense of entitlement, to control and dominate are defining features of IPV, as is the repetitive nature of the behavior and its tendency to escalate in severity [4].

Fatal outcomes of IPV include femicide, suicide, maternal mortality, abortion, stillbirth and AIDS. Non-fatal physical and mental health consequences of IPV are a matter of significant public health concern [5], [6]. Physical consequences include burns, fractures, chronic illness and pain syndromes, problems with hearing and sight, arthritis, seizures, headaches, sexually transmitted infections and HIV. Mental consequences include depression, anxiety, post traumatic stress, eating and substance use disorders. Strong links exist between IPV and risk of HIV infection [7], which is particularly relevant because South Africa has the largest number of people living with HIV/AIDS [8].

Despite the relevance of IPV to our burden of disease, health providers tend to resist identifying and managing IPV as a health issue [9]. In South Africa first contact care is mostly offered by primary care nurses with the support of doctors. Most guidelines recommend some form of screening or case finding in primary care that requires enquiry about problems with the relationship. There is also evidence that women appreciate being asked [10].

This study investigated how women with IPV present in primary care, how often IPV is recognized by health care practitioners and what other diagnoses are made. It also explored the experience of health practitioners who were expected to screen women for IPV. The results presented here are derived from a larger study that implemented and evaluated a screening and management protocol for IPV in primary care that was developed locally [9], [11]. The protocol recommended a universal approach to screening all women 18 years and older.

## Methods

Two urban and three rural community health centres (CHCs) in the Western Cape were purposefully selected as study sites according to the following criteria: typical of other CHCs in the Western Cape; having a mental health service; having a private room for the research assistant; having a sufficiently comprehensive service (radiological services, HIV testing and counseling).

The study aimed to identify 75 women per site or a total of 300 women to meet the requirements of the larger study. Four screening cycles were conducted for 4 to 8 weeks. Health care practitioners, namely all doctors and nurses at each site, were requested to screen all women 18 years and older using a menu of possible direct or indirect questions. In-service training of the health care practitioners was given by the researchers and they were provided with a prompt tool that listed the questions, such as, “How are things going in your relationship?” or “In this clinic we ask all women patients if they have ever experienced any form of abuse. Have you ever experienced abuse by your partner?” Those identified as having experienced IPV during the previous two years were referred to the study nurse on site, who then completed a modified management protocol with each participant [11].

The medical records of identified women were obtained and all consultations during the previous two years coded using the International Classification of Primary Care (ICPC-2) [12]. For each consultation the reason for encounter (RFE) and diagnoses were coded. ICPC-2 provides specific alpha-numeric codes for the commonest RFE and diagnoses in primary care. Inter-rater reliability was verified by an ICPC expert at Walter Sisulu University.

The ICPC coding was collated into SPSS and analyzed by the Centre for Statistical Consultation at Stellenbosch University. Most of the data was categorical in nature and analyzed in terms of frequency tables.

One focus group interview was held in the urban area with a male psychiatric nurse, four female clinical nurse practitioners and an emergency/trauma room nurse and a female doctor. One rural focus group interview was held with doctors and nurses who had referred women to the study and another focus group interview with those who had not. In both groups there was a spectrum of doctors from specialist family physicians to medical officers and psychiatric, maternity and primary care nurses of both genders. A final rural focus group interview included two lay counselors, a nurse manager and a clinical nurse practitioner, all female. Interviews explored their experience of screening and initial management of identified women. Key informant interviews were also conducted with the facility managers. Interviews were digitally recorded, transcribed verbatim and analyzed using the framework method [13].

The health care practitioner's written informed consent was obtained and the Health Research Ethics Committee of Stellenbosch University approved the investigation. The study was conducted in accordance with the principles of the Declaration of Helsinki.

## Results

### Women who had experienced IPV

One hundred and sixty eight women were identified with a history of IPV with 56 (33.3%) from the urban and 112 (66.6%) from the rural facilities. Overall the mean age was 36.7 years and women were mostly married (82, 48.8%), cohabiting (36, 21.4%) or currently single (35, 20.8%), and had a mean of 2.5 children (range 0–6 children).

In 54 cases the medical records were missing or the patient was attending the health centre for the first time. Therefore 114 medical records were entered into the final analysis. From these medical records, 1697 RFE were documented and 710 diagnoses made during the preceding two years, during which time these women were experiencing IPV and attending the health centre. The top 15 RFE and diagnoses are presented in Tables 1 and 2; and represent 53.1% and 59.4% of the total RFE and diagnoses. Only 11 of the 114 medical records (9.6%) demonstrated that the patient's difficulties with IPV had been previously identified. Recognition of the underlying issue of IPV was generally alluded to vaguely as “stress at home” or “social problem”.

**Table 1:** Reasons for encounter in women experiencing IPV.

Reasons for encounter (N = 1697)		n	%
1	Follow up for hypertension or heart problem (K64)	218	12.8
2	Headache (N01)	72	4.2
3	Request for psychiatric medication (P50)	66	3.9
4	Backache (L02, L03)	64	3.8
5	Follow up for diabetes (T64)	47	2.8
6	Sleep disturbance (P06)	42	2.5
7	Request for contraception (W50)	36	2.1
8	Feeling faint, giddiness, dizziness (N17)	28	1.6
	Sore throat/throat complaint (R21)	28	1.6
9	Cough (R05)	27	1.6
	Assault (Z25)	27	1.6
10	Fatigue/tiredness (A04)	23	1.4
	Feeling anxious/nervous/tense (P01)	23	1.4
	Follow up for psychiatric problem (P64)	23	1.4
	Pap smear (X37)	23	1.4
11	Nausea (D09)	21	1.2
	Feeling depressed (P03)	21	1.2
	Other psychological symptoms (P29)	21	1.2
	Bladder symptom/complaint (U13)	21	1.2
12	Request for anti-retroviral medication (B50)	19	1.1
13	Abdominal pain (D01, D06)	18	1.1
14	Painful respiration (R01)	17	1.0
15	Vaginal discharge (X14)	16	0.9

**Table 2:** Diagnoses in women experiencing IPV.

Diagnoses (N = 710)			
	Diagnosis	n	%
1	Depressive disorder (P76)	63	8.8
2	Uncomplicated hypertension (K86)	39	5.5
3	Sexually transmitted infection (A78)	27	3.8
4	Upper respiratory tract infection (R74)	25	3.6
5	Acute bronchitis (R78)	18	2.5
	Tuberculosis (A70)	18	2.5
6	Cystitis/urinary tract infection (U71)	17	2.4
	Asthma (R96)	17	2.4
7	HIV/AIDS (B90)	16	2.3
8	Pregnancy (W78)	15	2.3
9	Assault (Z25)	14	2.0
	Acute/chronic sinusitis (R75)	14	2.0
10	Vomiting (D10)	13	1.8
	Type 2 diabetes (T90)	13	1.8
11	Anxiety disorder/state (P74, P01)	12	1.7
12	Partner behavior problem (Z13)	11	1.5
13	Gastroenteritis, presumed infection (D73)	8	1.1
	Muscle pain (L18)	8	1.1
14	Tension headache (N95)	7	1.0
	Allergic rhinitis (R97)	7	1.0
	Boil/carbuncle (S10)	7	1.0
15	Constipation (D12)	6	0.8
	Vaginal discharge (X14)	6	0.8
	Syphilis (X70)	6	0.8

### Experience of the health care practitioners

The overall experience was that health care practitioners were reluctant to screen every patient and even frequent reminders and motivation from the researchers did not produce the participation of most. Facility managers revealed that IPV was often an issue in the personal lives of nurses and that this may have made it difficult for them to tackle the issue professionally:

*“As nurses you are taught to focus on other's needs and forget about your own … sometimes health care providers have this problem of being abused but they are quiet about it.”*

*“It's the shame – how will I come and work here again? Embarrassed … people mustn't know. Too private…if it's psychological issues nurses don't want to be patients because of stigma attached.”*

Health care practitioners saw IPV as a social and not a legitimate health problem. They were also concerned about having to deal with complex psychosocial issues, which could not easily be treated or fixed, when there were so many obvious biomedical problems.

*“It's not that we don't want to do it – it's something new … [we are] so used to examine, diagnose, medicate…and it opens up an area that is not easy to deal with…often an area that people struggle with: stress, psychosocial issues, messy…”*

*“To start off with something is sometimes very difficult. Something new is a challenge, it's also very intimidating.”*

*“At community health centres, health care providers are more inclined to focus on the physical…and don't feel comfortable with the emotional side of things…”*

Health care practitioners wanted women to leave the relationship and if they didn't then blamed the women for not taking immediate action. The benefit of recognizing these women was reduced by the perception that effective solutions were difficult to prescribe:

*“It's a whole anthill you are disturbing and maybe there are a lot of them who have had advice and help, but they just didn't want to help themselves…”*

*“I think if you have opened something up it should immediately be dealt with.”*

Health care practitioners who were dealing with a high workload were concerned at the additional time required to ask questions and to contain the issue of IPV once it was identified. One doctor was reluctant to ask again, after one patient started crying and took an extra 15 minutes:

*“…the most important thing was that there wasn't enough time to really ask these types of questions of the patients. We felt if you were going to do it for an extra 5–10 women per day…it would take close to an extra hour…many days we walk out of here at 18h30 so an extra hour is too much.”*

Some nurses who lived in the local community were reluctant to ask because it would be an invasion of privacy and nurses may be criticized for spreading stories. A few expressed concerns that they would be targeted by abusive partners:

*“Sisters don't want to screen because they know patients.”*

One doctor undergoing training in family medicine had more commitment to ask and found it easier as she persisted. One nursing sister, who was not from the local community, demonstrated commitment and success despite her high workload.

The spirit of the interaction and nature of the practitioner-patient relationship may also have impacted on the success of asking the question. Several practitioners complained patients did not disclose easily and seemed surprised at the sudden interest.

## Discussion

This study demonstrates that recognition of women with IPV is very low in South African primary care and adds useful new information on how women present to ambulatory health services. These findings offer key cues that can be used to improve case finding for IPV in resource-poor settings.

Women presented with a variety of RFE that should raise the index of suspicion for IPV: assault, headache, request for psychiatric medication, sleep disturbance, dizziness, fatigue, feeling anxious, depressed and other psychological symptoms. Low back pain and backache are also associated with psychosocial stress, especially when chronic [14]. None of the women presented the problem of IPV explicitly. Altogether 15% of the RFE listed in Table 1 were suggestive of mental problems indicating that IPV should be specifically asked about in patients presenting with psychological cues. In South Africa primary care providers rarely diagnose mental health disorders such as depression and anxiety, which makes the recognition of underlying IPV even less likely [15]. Furthermore, those already on psychiatric medication or diagnosed with a mental problem were not asked about IPV. Depression was the commonest diagnosis, but even in these patients, IPV was not identified as an important trigger. Many studies have shown a strong link between depression and IPV [16], [17]. Anxiety disorders were diagnosed occasionally and the literature suggests that these disorders are common amongst women with IPV [18], [19].

It is also clear from the RFE and diagnoses that women experiencing IPV are frequently attending for chronic diseases such as hypertension, asthma and diabetes. HIV/AIDS was the seventh most common diagnosis and evidence suggests that women who experience IPV are at increased risk of HIV [20]. Increased psychosocial stress from IPV may negatively impact on adherence to medication, self-care and control of chronic disorders. The diagnosis of sexually transmitted infections was twice as common in this group of women, compared to primary care as a whole [15]. Opportunities for identification of women with IPV were evident in requests for contraception, cervical smears and pregnancy-related consultations.

Interestingly assault was not the most common RFE, although this would be the most obvious prompt to asking about IPV, and the majority of women would have been missed if this was the only identifying feature. Nevertheless assault was three times as common an RFE in this sub-group of women compared to primary care as a whole [15].

The literature also suggests that alcohol or substance abuse is an important issue [21], [22]. This did not appear in the RFE or diagnoses, but in the larger study 18.5% of the women admitted to an alcohol problem [9].

Universal screening, although recommended in the South African protocol, was difficult to implement in practice. This resistance stemmed from concerns about opening a ‘Pandora's box’ of time-consuming social and psychological problems that health care practitioners felt ill-equipped to deal with. There was also resistance to seeing IPV as a legitimate medical problem. Practitioners became frustrated with women who were not easy to “fix” with medication and who did not readily follow instructions to leave the relationship. Practitioners may become dismissive of women who do not follow their advice. Many practitioners appeared to be too close to the issue in their personal lives or social network and needed to distance themselves emotionally [23]. IPV was treated as an acute problem that required an effective treatment, rather than as a chronic problem with the need for support through a process of decision making and behavior change.

Given the potential cues presented by patients with IPV and the experience of trying to implement universal screening we would argue that selective case finding of women with a higher risk of IPV would be a more constructive approach in resource-poor settings.

Although the study only evaluated records from five purposefully selected primary care facilities, the rural/urban mix makes it likely that the results obtained are fairly typical of primary care facilities in the Western Cape. It is possible that IPV was recognized by primary care providers, but not recorded as a diagnosis in the medical record. However this would also imply that the diagnosis was not seen as important enough to record. The exact relationship between RFE and diagnoses obtained and IPV is not possible to determine in this study. However the differences between these women's RFE and diagnoses and those of women presenting to primary care in general have been discussed and interpreted. It is possible that health care practitioners were less positive about screening than the findings suggest as there could be some obsequiousness bias within the interviews.

The study has implications for the training of health care practitioners in the district health services. Clinical nurse practitioners, medical officers and family physicians should be trained to associate typical RFE with the possibility of IPV. Furthermore, training should enable them to instigate a comprehensive biopsychosocial management plan that includes attention to women's legal rights. Increasing the ability of practitioners to recognize and assess mental disorders is also linked to IPV. Training should recognize the need to help practitioners who themselves experience IPV and to explore attitudes and norms regarding gendered human rights.

A model for the management of IPV in South African primary care is referred to elsewhere [24]. This model only requires the practitioner to screen for IPV and attend to relevant clinical matters before referring to an ‘IPV champion’ who will provide a more comprehensive assessment and management plan.

### Conclusion

Less than 10% of women experiencing IPV are recognized in South African primary care. The following cues in the patient's history should enable selective case finding for IPV: sexually transmitted infections including HIV, assault, chronic pain syndromes and symptoms suggestive of a mental problem e.g. sleep disturbance, tiredness, feeling depressed or anxious; history of a mental illness or psychiatric medication.

Opportunistic case finding for IPV should also be made routine in patients attending for family planning, cervical smears and antenatal care as well as for non-communicable chronic diseases, HIV/AIDS and TB. Attention to recognition and management of women experiencing IPV should be increased in the training of primary care providers.
